# Evaluation of ThinkQA (v2.0.1.11) as an online secondary dose check for MR guided radiation therapy with the Elekta Unity MR-Linac

**DOI:** 10.1007/s13246-025-01693-0

**Published:** 2026-01-19

**Authors:** Ariadne S. Brodmann, John A. Baines

**Affiliations:** 1 Department of Medical Physics, Townsville University Hospital and Health Service, Townsville, QLD 4814 Australia; 2 College of Science and Engineering, James Cook University, Townsville, QLD 4811 Australia

**Keywords:** MR-Linac, Secondary dose calculation, Adaptive planning, Radiotherapy

## Abstract

To evaluate ThinkQA (TQA), a collapsed cone convolution–based secondary dose check, as an alternative to MU2net (Clarkson-based, point-dose) for online adaptive planning on the Elekta Unity 1.5 T MR-Linac at Townsville University Hospital. Commissioning followed MPPG 5.b tests. The reference-dose agreement, magnetic-field modelling, directional dependence, output factors, off-axis points, heterogeneous slab geometries and calculation properties were assessed. Nine step-and-shoot IMRT plans (courtesy of Elekta) and 226 retrospectively analysed adapted fractions (prostate and pelvic nodes; planning target volumes 1.9–170.0 cm^3^) were compared between TQA and Monaco by gamma analysis (global 10.0% threshold; 2.0%/2.0 mm, 3.0%/2.0 mm). At 10.0 cm depth under TQA reference conditions, the mean absolute point-dose difference versus Monaco was 0.4%. TQA reproduced models the magnetic-field–induced cross-plane asymmetry with close agreement to Monaco. Directional dependence differences were ≤  ± 1.2% except when traversing the couch (± 1.8%). Output factors agreed within ≤ 1.0% (SSD 133.5 cm) and ≤ 2.0% (SSD 138.5 cm). In 226 clinical fractions, 3.0%/2.0 mm (global) yielded 93.0% passes in the high-dose region and 100.0% in other regions; 2.0%/2.0 mm yielded 25% high-dose passes. TQA results were available within about 1 min post Monaco export. TQA provides accurate, rapid, volumetric secondary dose verification for Unity, improves agreement with Monaco, and reduces console time by eliminating dose point re-selection. A 3.0%/2.0 mm global gamma criterion is a clinical acceptance level, with tighter criteria reserved for targeted investigations.

## Introduction

MR-guided linear accelerators (MR-Linac) have been described extensively, highlighting their ability to combine magnetic resonance imaging with radiotherapy delivery to enable online adaptive radiotherapy [1–5]. The Elekta Unity MR-Linac (Elekta AB, Stockholm, Sweden) system referenced in this work integrates a 1.5 T MRI scanner (modified Ingenia MR scanner, Philips, Netherlands) with a 7 MV flattening filter-free linac, allowing real-time imaging and adaptive radiotherapy planning using Graphical Processing Unit (GPU) based Monte Carlo treatment planning (Monaco v.5.4) [6–8]. The Monte Carlo dose calculation algorithm (GPUMCD) incorporates radiation transport in the presence of a magnetic field and utilizes GPU technology to enable parallel processing, significantly reducing calculation times [9]. The Unity is a perpendicular MR-Linac system where the beam direction (IEC61217 X–Z plane) is orthogonal to the static magnetic field B_0_ (IEC61217 Y direction).

Currently supported treatment techniques include conformal therapy and step-and-shoot IMRT (SSIMRT). The adaptive SSIMRT planning options available with the Monaco treatment planning system (TPS) used with the Elekta Unity are broadly categorized into two workflows: Adapt to Position (ATP) and Adapt to Shape (ATS). ATP primarily accounts for daily variations in patient positioning by applying rigid transformations, whereas ATS facilitates recontouring of targets and organs-at-risk (OAR) to account for anatomical variations in their shape and location. For a detailed description of these adaptive treatment options and the associated online calculation of adapted treatment plans the reader is referred to Winkel et al. [2].

Conventional pre-treatment patient specific quality assurance (PSQA) measurements are not feasible for a given adapted plan in a daily treatment workflow. Albeit that PSQA can be performed post treatment at the clinics discretion it is essential to have an independent, online dosimetric verification for each adapted plan–commonly known as a secondary monitor unit (MU) or secondary dose check (SDC)–before clinical delivery [10].

Commercial SDC solutions range from point-dose comparison using simplified Clarkson integration (MU2net, DOSIsoft, France) to volumetric dose computation (RadCalc, Lifeline Software, USA) [11, 12]. Several groups have used collapsed-cone convolution (CCC) based planning systems such as OnCentra (Elekta), and RayStation (Raysearch Laboratories, Stockholm, Sweden) as independent MU checks for Elekta Unity. Albeit that these systems do not model the impact of B₀ on dose deposition, reducing accuracy at 1.5 T [13, 14]. Other groups modelled the influence of B₀ either with MC electron transport in the presence of 0.35–1.5 T magnetic fields [8, 15] or via manual/approximate corrections, such as a modified Clarkson method including B_0_ field terms [16] or geometric adjustments to jaws/MLC to emulate 1.5 T behaviour [12]. A finite-size pencil-beam algorithm with a Lorentz-force correction for the magnetic field (ClearCalc, Radformation Inc.) has also been reported [17]. While these strategies improve accuracy, they are typically computationally slower or operationally burdensome, limiting their practicality for fast online verification.

In 2019, when the Elekta Unity was introduced clinically at the Townsville University Hospital (TUH), MU2net (DOSIsoft, France) was employed as a secondary dose check [18]. Although MU2net accounted for the magnet cryostat of the Unity, due to its simplistic Clarkson-based algorithm significant time pretreatment was required to select dose reference points (DRPs), (up to 1 h for a 9-field prostate SSIMRT plan) for MU2net and Monaco that agreed to within 5%. Due to a limitation of Monaco, only one DRP per beam could be selected and at treatment it was common that DRPs for a given beam of an adapted plan would differ by  < 5.0%. In such cases a new DRP would be selected in Monaco for each of the beams concerned. Subsequently the CT data set, RT Plan and RT structure set was re-exported to MU2net for an additional DRP intercomparison. Particularly for small lesions (PTV < 7.0 cc) it was often necessary to repeat this process several times until DRP discrepancies were within tolerance, adding to patient treatment time. Often up to 10 min re-calculation time was necessary.

In 2024, TUH introduced ThinkQA v2.0.1.11 (TQA), the latest SDC software from DOSIsoft and the commissioning of this SDC is the subject of this work.

TQA is a substantial improvement compared to MU2net, employing a CCC algorithm and modelling of the attenuation of the cryostat and posterior coil of the MRI system. In addition, TQA calculates a volumetric dose distribution in the presence of the static 1.5 T magnetic field environment of the Unity.

In the Unity adaptive workflow TQA SDC imports DICOM datasets (planning CT, RT Structure Set, RT Plan, RT Dose) from the primary TPS adapted plan. Dose calculation involves two sequential steps: beam fluence reconstruction at the treatment head exit and medium-specific dose computation. A multi-source photon beam model calculates the photon fluence, incorporating primary and extra-focal sources. The fluence model accounts for beam-specific components such as the MLC configuration, and uniquely for Unity, the beam transmission through the liquid helium-filled magnet cryostat. The CCC algorithm employs pre-calculated energy deposition kernels to efficiently model photon interactions and energy transport in heterogeneous patient anatomy. The kernels (“warped kernels”) reflect the dose deposition resulting from magnetic field-electron interactions due to the Lorentz force. Although the electron return effect (ERE) at air-tissue interfaces is significant, ERE is not explicitly modelled in TQA, causing known dosimetric deviations approximately 10.0 mm either side of heterogenous interfaces [19]. TQA-SDC translates CT-based Hounsfield Units into relative electron densities and applies voxel-based dose conversion factors, enabling direct comparisons between CCC dose-to-water and Monte Carlo-reported dose-to-medium [19].

**Fig. 1:**
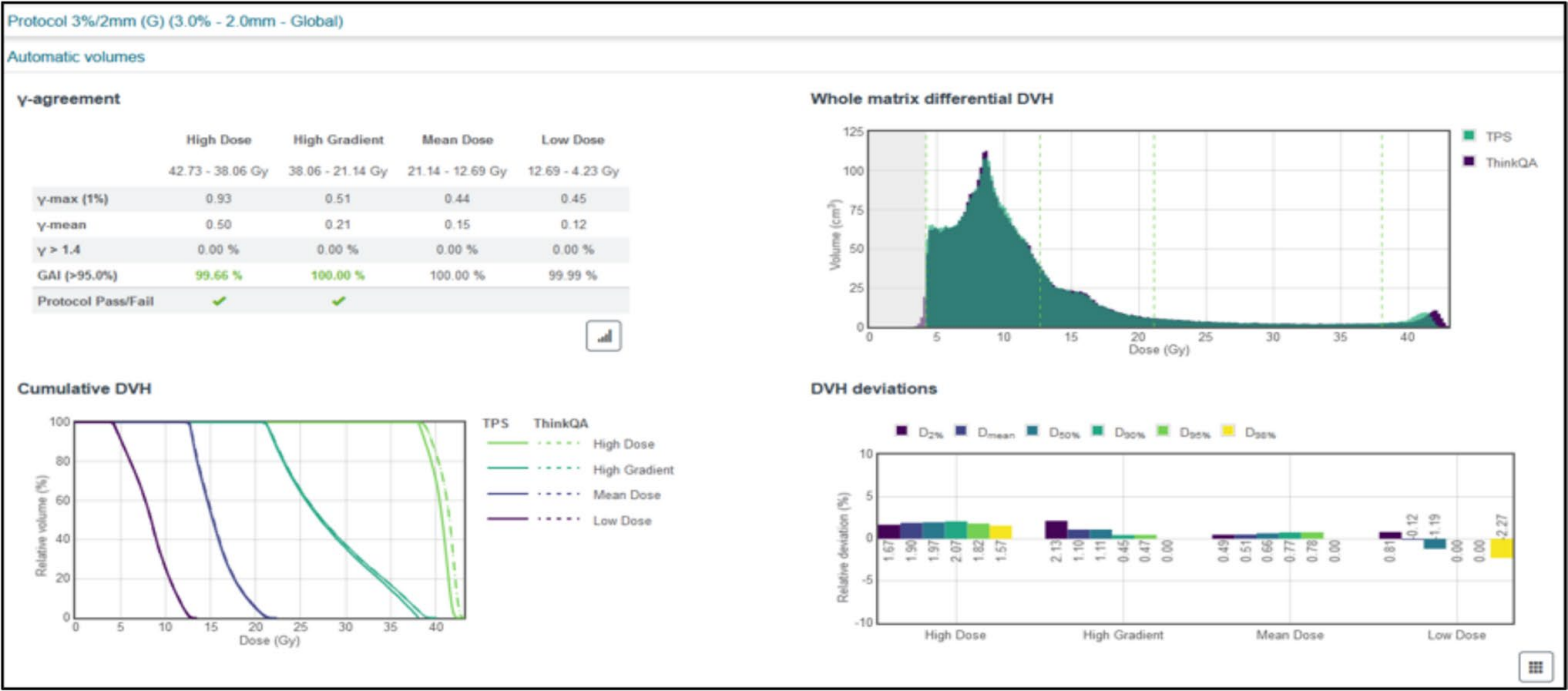
Screenshot of TQAs overall volume gamma results protocol interface (DOSIsoft, France)

TQA (Fig. 1) reports gamma analysis across four discrete dose regions: high dose (HD, maximum dose to 90.0%), high gradient (HG, 95.0–50.0%), mean dose (MD, 50.0–30.0%), and low dose (LD, 30.0–10.0%), assessing planning target volumes (PTVs), OAR interfaces, and general OAR and healthy tissues respectively. TQA also provides differential and cumulative dose-volume histogram (DVH) statistics and region-specific gamma evaluations [20].

The clinical implementation and validation of TQA at TUH involved two phases: commissioning of the CCC algorithm following Medical Physics Practice Guideline 5.b (MPPG5b) recommendations (including but not exclusively output factors, directional dependence, off-axis fields, and heterogeneity scenarios) [21]; and clinical gamma comparison assessing the impact of Monaco calculation parameters with nine SSIMRT beam model verification plans (courtesy of Elekta) and retrospective analysis of clinically delivered treatments.

To the best of our knowledge this is the first report dedicated to implementing TQA v2.0.1.11 software within the Elekta Unity adaptive planning workflow. For review of TQA v2.0.0.60 the reader is referred to Rojas–López et al. [22].

## Materials and methods

A CT dataset was acquired for solid water phantoms with dimensions 30.0 cm × 30.0 cm × 19.0 cm and 30.0 cm × 30.0 cm × 24.0 cm. The data sets were acquired on a large bore CT scanner (Canon Aquilion LB), using a pelvis protocol (300 mA, 120 kV, 3.0 mm slice thickness 550.0 mm field of view) and imported into Monaco for dose computation with various beam configurations and SSIMRT plans as described below. The phantom dimensions (z-direction) 19.0 cm and 24.0 cm enable Monaco calculations of dose at a depth of 5.0 cm and 10.0 cm. This configuration is adopted since the isocentre of the Unity is 14.0 cm (FAD = 143.5 cm) above the couch top in the bore of the Unity which is relevant for our clinical film PSQA that utilises similar solid water stacks.

Unless otherwise stated, all dose computations were simulated in Monaco Unity v5.4 using the following calculation properties: 0.1 cm grid size, 0.5% statistical uncertainty (SU) per control point (cp), dose deposition to medium and a phantom material lookup table. For each plan, the RT Dose, RT Structure and CT dataset were exported to TQA v2.0.1.11 for dose calculation and subsequent gamma analysis.

For comparison of percentage depth dose (PDD), profiles and point doses (0.15 cc) for TQA and Monaco, the DICOM data for each associated beam was exported from TQA. Using an in-house script, PDDs, profiles and point doses were extracted from the DICOM data and compared with corresponding Monaco data.

For gamma comparison, a gamma pass rate of ≥ 95.0% was applied consistent with clinical practice at our facility. Unless otherwise stated gamma comparisons used a 10.0% dose threshold with a global gamma normalisation.

## Reference dose validation

The absolute dose output calculated by TQA and Monaco were compared using TQA’s specified reference conditions: 1.0 Gy / 100 MU (B_0_ = 1.5 T), depth 10.0 cm, at isocentre (143.5 cm), 133.5 cm SSD, gantry 0.0°, for a 10.0 cm × 10.0 cm field. It should be noted that Monaco uses different reference conditions: 1.0 Gy/100 MU (B_0_ = 1.5 T) at the isocentre, depth of 5.0 cm in water, 138.5 cm SSD, gantry 90.0°, for a 10.0 cm × 10.0 cm field. To facilitate direct comparison, TQA’s reference conditions were replicated in Monaco and dose was calculated, using a SU of 0.1%. At TUH the Elekta Unity reference conditions are the same as Monaco’s. These conditions were also computed in TQA to enable direct comparison.

### TQA modelling of dose deposition due to B_0_

To validate the modelling of the static magnetic field (B_0_ = 1.5 T) on dose distribution, TQA and Monaco cross-plane (IEC61217 X-axis) and in-plane (IEC61217 Y-axis) profiles at 10.0 cm depth (133.5 cm SSD) were compared for a 2.0 cm × 2.0 cm, 10.0 cm × 10.0 cm and 22.0 cm × 22.0 cm field.

### Directional dependence

A cylindrical phantom (6.0 cm diameter, 21.0 cm length, forced RED = 1.000) was simulated in Monaco. The long axis of the phantom was aligned with the Y-axis and dose was calculated at the isocentre on the central axis of the cylinder for a 5.0 cm × 5.0 cm field and gantry angles ranging from 0.0 to 120.0º and 225.0 to 360.0º in 15.0º increments and 120.0 to 225.0º in 5.0º increments (beams traversing the couch). DICOM data for each beam was exported to TQA and isocentric doses were calculated for comparison with corresponding Monaco beams. For comparison TQA and Monaco doses were normalised to their respective output values at gantry 90.0º.

Measurements on the Elekta MR Unity were acquired using a PTW30013 Farmer chamber (SN: 011298) and PTW Webline electrometer (SN: 00017), with the chamber positioned along the long axis of an in-house water-filled rotatable cylindrical phantom (6.0 cm diameter, 21.0 cm length). Measurements were obtained for gantry angles ranging from 0.0 to 360.0º. The phantom was rotated about the central axis in 15.0° increments to maintain consistent chamber orientation relative to the radiation source (results have been previously published [18]).

### Output factors

TQA and Monaco output factors (OFs) were calculated for central axis fields ranging in size from 1.0 cm × 1.0 cm to 20.0 cm × 20.0 cm at depths of 5.0 cm (138.5 cm SSD) and 10.0 cm (133.5 cm SSD). All OFs were normalised relative to a 10.0 cm × 10.0 cm reference field.

### Off-axis fields

TQA and Monaco point doses for off-axis square fields were compared between. Fields sizes1.0 cm × 1.0 cm, 3.0 cm × 3.0 cm and 5.0 cm × 5.0 cm at 138.5 cm SSD were evaluated, with field centres positioned every 2.5 cm between X = ± 12.5 cm, Y = ± 7.5 cm and at diagonal positions (± 10.0 cm, ± 7.5 cm).

### Water-lung and water-bone interfaces

To evaluate the effect of the absence of the ERE modelling in TQA, dose distributions in a heterogeneous slab geometry were simulated for field sizes of 1.0 cm × 1.0 cm and 5.0 cm × 5.0 cm. Each beam was incident normally on a phantom consisting of a 3.0 cm water (forced RED = 1.000), 3.0 cm of either cortical bone (forced RED = 1.707) or lung (forced RED = 0.306), followed by 14.0 cm of water (forced RED = 1.000). For each field size and slab composition PDDs on the central axis were calculated and percentage differences between TQA and Monaco were evaluated.

### Calculation properties

The effects of varying dose calculation grid spacing and SU on gamma analysis results for Monaco-TQA comparisons were investigated for a clinical prostate plan. This plan was recalculated in Monaco using combinations of grid sizes (0.1 cm, 0.2 cm, 0.3 cm) and SU per cp (1.0%, 2.0%, 3.0%). For each combination DICOM data was exported from Monaco to TQA and dosimetric comparisons were obtained using clinical gamma criteria of 2.0%/2.0 mm and 3.0%/2.0 mm.

### Sample IMRT plans

Nine SSIMRT plans (courtesy of Elekta) were simulated in Monaco using a homogeneous slab phantom (30.0 cm × 30.0 cm × 19.0 cm, forced RED = 1.000). Monaco, dose calculations were performed with a grid spacing of 0.2 cm and a SU of 3.0% per cp following our clinical protocol. DICOM data sets were exported to TQA and dosimetric comparisons of corresponding dose distributions were performed using gamma criteria of 1.0%/1.0 mm, 2.0%/1.0 mm and 2.0%/2.0 mm. For independent verification, each SSIMRT plan was delivered to Gafchromic™ EBT3 film (film placed at 5.0 cm depth) and compared to Monaco dose maps using gamma analysis with clinical criteria of 2.0%/2.0 mm. All comparisons returned > 95% gamma pass rates.

### Clinical cases

A retrospective gamma analysis review was performed on 226 treated clinical fractions to evaluate the correspondence of TQA and Monaco dose maps using clinical gamma criteria 2.0%/2.0 mm and 3.0%/2.0 mm.

Common treatment sites analysed included intact prostate, common iliac node, external iliac node, pelvic node and seminal vesicles (SV), with PTV sizes ranging from 1.9 to 170.0 cm^3^.

To visualise regions with dose discrepancies, TQA calculated dose distributions were exported and compared to Monaco calculated doses using external analysis software, VeriSoft v7.2 (PTW Freiburg GmbH, Freiburg, Germany). Gamma analyses were performed in sagittal, coronal, and transverse planes through the isocentre, and failing points on isodose maps were generated. Comparisons were performed using gamma criteria 1.0%/1.0 mm (local), 2.0%/2.0 mm (local), and 3.0%/2.0 mm.

All 226 fractions had previously undergone clinical PSQA using either Gafchromic™ EBT3 (for maximum doses < 10.0 Gy) or EBT-XD film (for maximum doses > 10.0 Gy) (Ashland, USA). Film-Monaco gamma analysis achieved > 95% pass rates using 2.0%/2.0 mm. Film analyses were performed in FilmQA Pro™ (Ashland, USA) using triple-channel analysis [23].

## Results

### Reference dose validation

Under TQA reference conditions, at 10.0 cm depth, the mean absolute point dose difference between TQA and Monaco was 0.4%, which is within the recommended tolerance outlined by MPPG5b [21]. Under the Elekta Unity calibration conditions (1.0 Gy/100 MU at 5.0 cm depth), TQA calculated a 1.2% difference relative to the nominal 1.0 Gy.

### TQA modelling of dose deposition due to B_0_

**Fig. 2:**
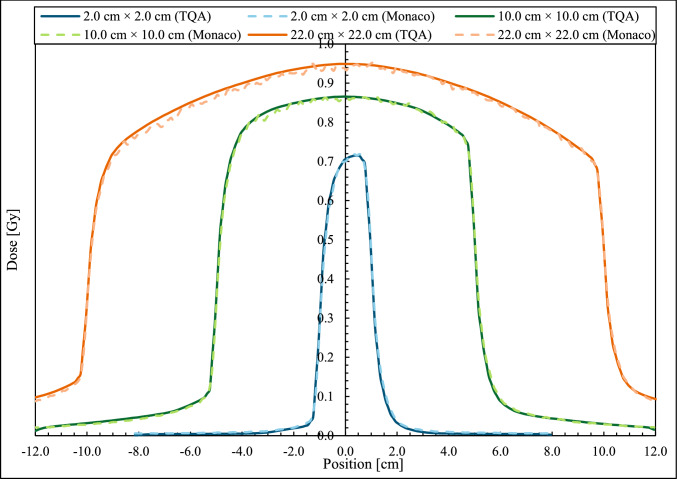
Cross-plane profiles for TQA and Monaco for a 2.0 cm × 2.0 cm, 10.0 cm × 10.0 cm and 22.0 cm × 22.0 cm field at 10.0 cm depth (133.5 cm SSD)

**Fig. 3:**
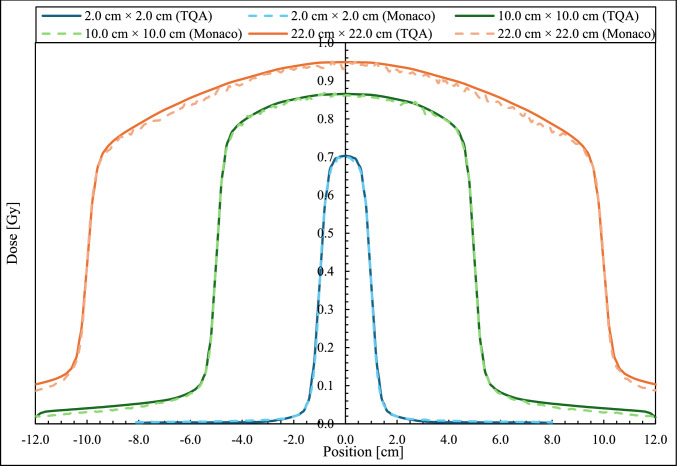
In-plane profiles for TQA and Monaco for a 2.0 cm × 2.0 cm, 10.0 cm × 10.0 cm and 22.0 cm × 22.0 cm field at 10.0 cm depth (133.5 cm SSD)

TQA reproduced the characteristic magnetic-field–induced asymmetry in the cross-plane (IEC61217 ± X direction, perpendicular to B_0_) (Fig. 2), and is symmetrical in the in-plane direction (IEC61217 ± Y direction, parallel to B_0_) (Fig. 3) [24]. For field sizes up to 10.0 cm × 10.0 cm, profiles showed good agreement with Monaco. For the 22.0 cm × 22.0 cm field, small discrepancies relative to Monaco were observed.

### Directional dependence

**Fig. 4:**
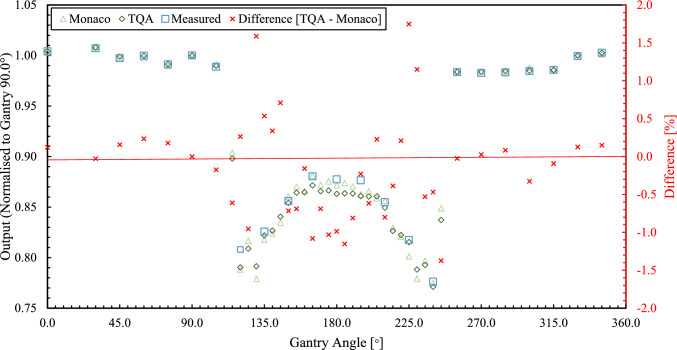
Directional dependence of output with gantry angle for 5.0 cm × 5.0 cm field, between Monaco, TQA and Measured. Percentage differences displayed are between TQA and Monaco

Measured, Monaco and TQA output values as a function of gantry angle, normalised to gantry 90.0°, are shown in Fig. 4. The largest deviation observed between TQA and Monaco is 1.8% at gantry 225.0°, which corresponds to the beam traversing the side of the treatment couch. For gantry angles 120.0 to 240.0° (beams traverse the couch), differences ranging from 1.8 to − 1.2% are observed. A variation of − 1.4% is observed at gantry angle 245.0°. Deviations remained within ± 1.2% for all gantry angles not traversing the couch.

### Output factors

**Fig. 5:**
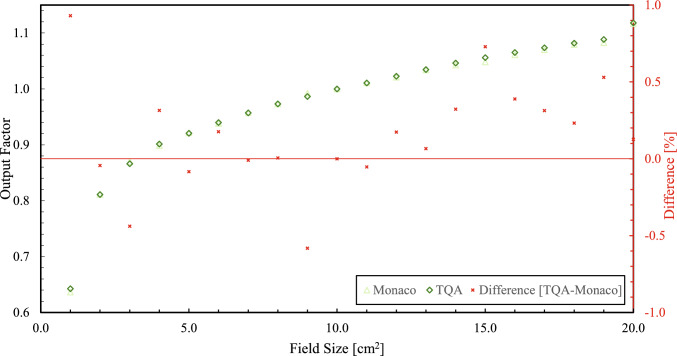
OFs for Monaco and TQA at 133.5 cm SSD. Percentage differences displayed are between TQA and Monaco

**Fig. 6:**
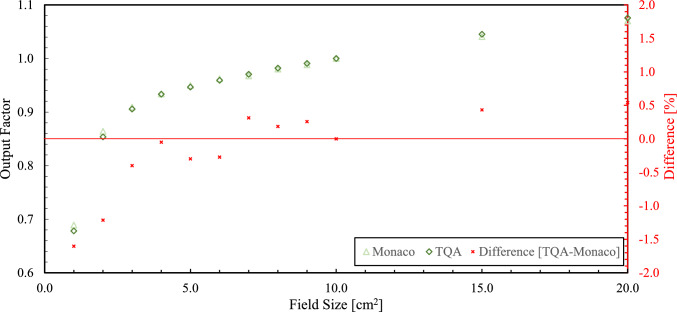
OFs for Monaco and ThinkQA at 138.5 cm SSD. Percentage differences displayed are between TQA and Monaco

OFs for TQA and Monaco, for field sizes of 1.0 cm × 1.0 cm to 20.0 cm × 20.0 cm at 133.5 cm SSD (10.0 cm depth) and 138.5 cm SSD (5.0 cm depth), are shown in Fig. 5 and Fig. 6, respectively. At SSD 133.5 cm, for all field sizes OFs were within 1.0%, with the greatest deviation (0.9%) observed for a 1.0 cm × 1.0 cm field. At SSD 138.5 cm OF differences for field sizes from 3.0 cm × 3.0 cm to 20.0 cm × 20.0 cm were within 0.6%, discrepancies increased for smaller fields, reaching − 1.2% for 2.0 cm × 2.0 cm and − 1.6% for 1.0 cm × 1.0 cm. All results where within the recommended ± 2.0% tolerance [21].

### Off-axis fields

**Fig. 7:**
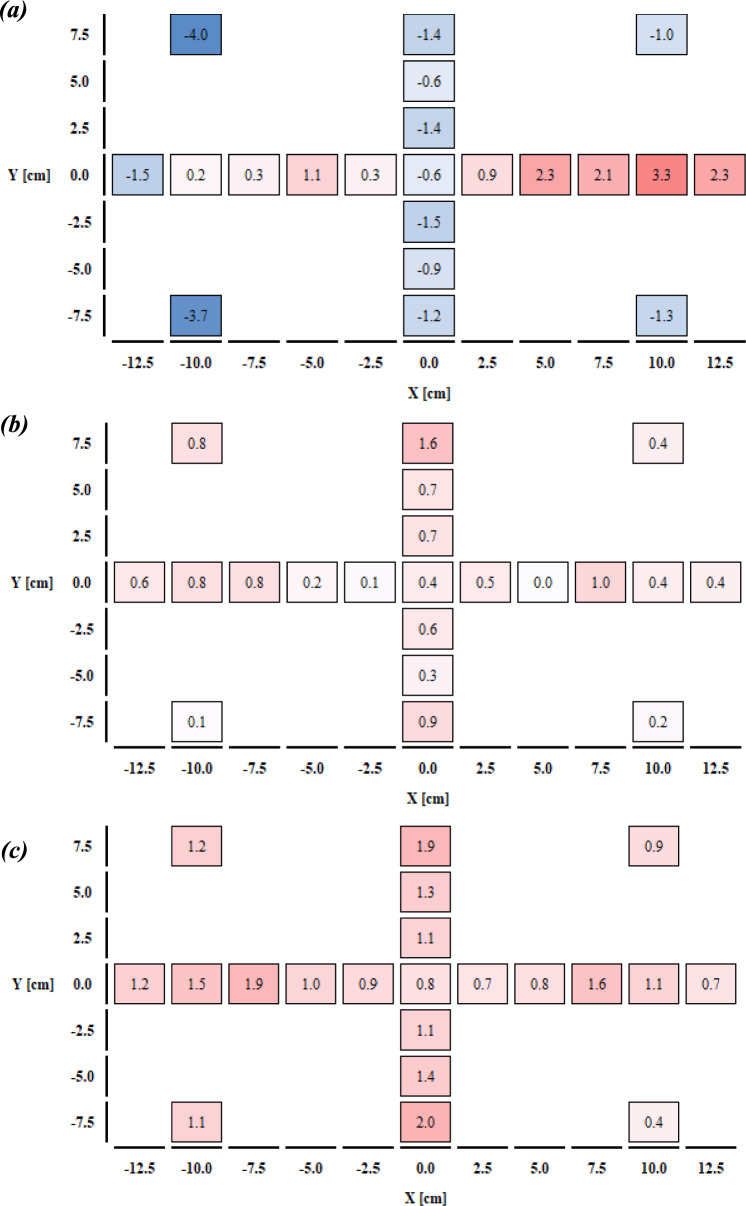
Off-axis point dose differences between TQA and Monaco for the following field sizes; **a** 1.0 cm × 1.0 cm, **b** 3.0 cm × 3.0 cm and **c** 5.0 cm × 5.0 cm. The axis spans from − 12.5 to 12.5 cm in the lateral (X-axis) direction and from − 7.5 to 7.5 cm in the vertical (Y-axis) direction. Percentage dose differences are represented on a colour scale, ranging from positive values (red) to negative values (blue)

Off-axis point dose differences between TQA and Monaco for field sizes (a) 1.0 cm × 1.0 cm, (b) 3.0 cm × 3.0 cm and (c) 5.0 cm × 5.0 cm at various positions are shown in Fig. 7.

The smallest field size (1.0 cm × 1.0 cm, Fig. 7a), returned the greatest discrepancies, with differences ranging from − 4.0 to + 3.3%. Significant negative differences were observed along the Y-axis, including the diagonally positioned points.

For the intermediate field size (3.0 cm × 3.0 cm, Fig. 7b), agreement improved, with the majority of differences being within ± 1.0%, apart from a single outlier of + 1.6% at [0.0, 7.5]. The magnitude and spatial distribution of deviations were more symmetric compared to the smallest field.

For the largest field size (5.0 cm × 5.0 cm, Fig. 7c), dose discrepancies were within ± 2.0%, with the largest deviations occurring further from the central axis at position: [0.0, − 7.5].

### Heterogeneity

**Fig. 8:**
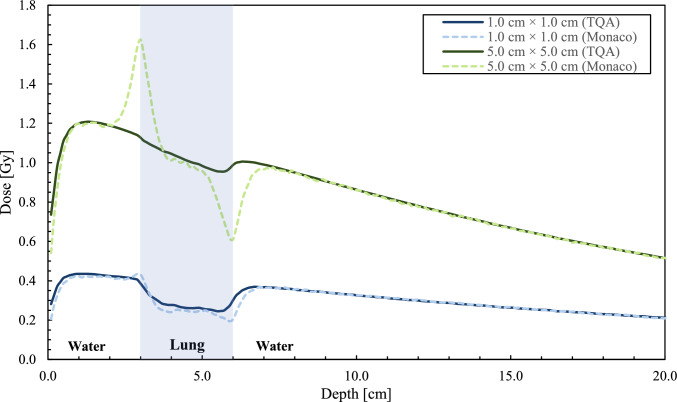
PDD curves through a 3.0 cm lung-equivalent slab (RED = 0.300) for field size of 1.0 cm × 1.0 cm and 5.0 cm × 5.0 cm

**Fig. 9:**
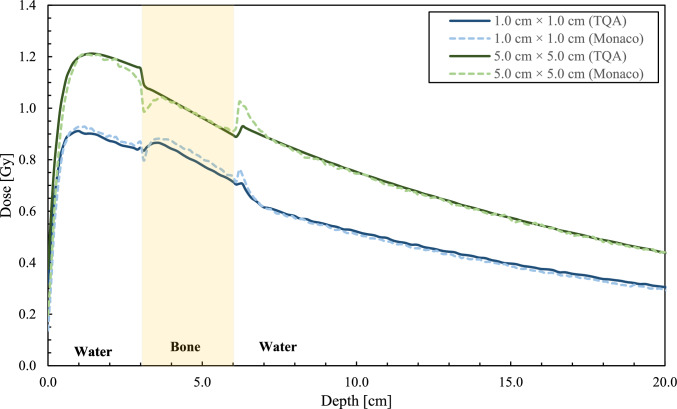
PDD curves through a 3.0 cm cortical bone-equivalent slab (RED = 1.700) for field size of 1.0 cm × 1.0 cm and 5.0 cm × 5.0 cm

Figures 8 and 9 display the calculated depth-dose curves for TQA and Monaco for 1.0 cm × 1.0 cm and 5.0 cm × 5.0 cm field sizes normally incident on a composite water–lung–water and water–cortical bone–water slab geometry, respectively. These profiles highlight the influence of tissue heterogeneities on photon beam attenuation and dose distribution at interfaces.

For the water-lung-water slab, both Monaco and TQA demonstrate close agreement in the build-up region. Deviations become apparent approximately 0.8 cm before the water–lung interface and continue until about 1.0 cm beyond it. Within the bulk of lung region, TQA and Monaco are in good agreement for both field sizes and significant differences are evident at the interface regions for the 5.0 cm × 5.0 cm field. For the smaller field discrepancies are observed closer to the interface, occurring approximately 0.3 cm before and 0.1 cm after the interface and with reduced differences comparted to the 5.0 cm × 5.0 cm field. Beyond the lung–water interface region, TQA and Monaco are in good agreement for both field sizes.

In the cortical bone-equivalent slab, TQA and Monaco similarly demonstrate good agreement away from the interface regions and within the bulk of the bone slab. At the water–bone and bone-water interfaces dose discrepancies between TQA and Monaco are observed with Monaco showing a dose fall and rise at the former and a dose rise and fall at the latter. Such trends are also observed at the lung-water interface (lower density to higher density) and water-lung interface (higher density to lower density). Deviations between TQA and Monaco are evident about 0.8 cm before the water–bone interface and persist approximately 1.0 cm beyond it. For the smaller field size, smaller discrepancies are evident at both interfaces, and Monaco simulates a slightly higher dose within the cortical bone region. Differences between TQA and Monaco continue up to approximately 0.5 cm beyond the distal interface.

### Calculation properties

For the clinical plan investigated TQA-Monaco gamma agreement index (GAI) results, criteria 3.0%/2.0 mm, for a given SU are approximately independent of grid size for all dose regions (Table 1). At 2.0%/2.0 mm grid size does affect the gamma values for the HD region and changing the grid size from 0.2 to 0.3 cm results in a maximum difference of 2.2% in this region, with minimal variation observed across other regions. For a given grid size changing the statistical uncertainty from 1.0 to 3.0% significantly affects the gamma value, showing up to a 7.3% difference (grid size 0.1 cm) in the HD region.

**Table 1:** TQA gamma analysis (criteria: 2.0%/2.0 mm and 3.0%/2.0 mm) for various combinations of grid sizes and SU/cp. Italics: 100.0%–95.0%, Bold: 95.0%–90.0% and Bold italics: < 90.0%

Gamma criteria		2.0%/2.0 mm				3.0%/2.0 mm			
Grid size [cm]	SU per cp [%]	GAI (> 95.0%)				GAI (> 95.0%)			
		HD	HG	MD	LD	HD	HG	MD	LD
0.3	1.0	**90.2**	*99.9*	*99.6*	*98.0*	*99.4*	*100.0*	*99.8*	*98.8*
	2.0	***86.5***	*99.9*	*99.7*	*98.0*	*98.6*	*100.0*	*99.9*	*98.9*
	3.0	***84.1***	*99.9*	*99.5*	*98.0*	*97.0*	*100.0*	*99.9*	*98.8*
0.2	1.0	***88.9***	*99.8*	*99.7*	*98.0*	*99.3*	*100.0*	*99.9*	*98.8*
	2.0	***86.0***	*99.8*	*99.6*	*98.0*	*98.4*	*100.0*	*99.9*	*98.8*
	3.0	***81.9***	*99.7*	*99.4*	*97.9*	*96.0*	*100.0*	*99.9*	*98.8*
0.1	1.0	**90.3**	*99.8*	*99.7*	*97.9*	*99.4*	*100.0*	*99.9*	*98.7*
	2.0	***86.7***	*99.8*	*99.6*	*97.9*	*98.3*	*100.0*	*99.9*	*98.7*
	3.0	***82.9***	*99.6*	*99.4*	*97.9*	*96.3*	*99.9*	*99.9*	*98.7*

### Sample IMRT plans

Gamma analysis for nine SSIMRT plans (courtesy of Elekta) are presented in Table 2. All plans, across all regions, returned gamma > 95.0% at 2.0%/2.0 mm, with all but the lung plan maintaining pass rates > 95.0% at a stricter criterion of 2.0%/1.0 mm. Generally, all plans exhibit gamma pass rates < 95.0% at 1.0%/1.0 mm across various dose regions.

**Table 2:** TQA gamma analysis (criteria: 1.0%/1.0 mm, 2.0%/1.0 mm and 2.0%/2.0 mm) for 9 × SSIMRT plans supplied courtesy of Elekta, Italics: 100.0%–95.0%, Bold: 95.0%–90.0% and Bold italics: < 90.0%

Plan ID	Gamma criterion (mm)	GAI (> 95.0%)			
		HD	HG	MD	LD
Prostate 1	2.0%/2.0	*99.7*	*99.4*	*99.2*	*97.4*
	2.0%/1.0	*97.6*	*98.3*	*98.6*	*96.6*
	1.0%/1.0	***79.0***	***85.3***	**94.1**	**92.7**
Prostate 2	2.0%/2.0	*99.8*	*99.7*	*100.0*	*98.4*
	2.0%/1.0	*98.9*	*99.2*	*99.9*	*97.7*
	1.0%/1.0	***85.5***	**92.8**	*96.6*	*95.2*
Prostate 7	2.0%/2.0	*99.5*	*100.0*	*99.7*	*98.0*
	2.0%/1.0	*98.9*	*100.0*	*99.4*	*96.9*
	1.0%/1.0	***79.3***	*98.2*	*96.4*	**93.8**
Prostate 9	2.0%/2.0	*99.9*	*99.9*	*98.6*	*98.9*
	2.0%/1.0	*99.9*	*99.7*	*97.7*	*98.3*
	1.0%/1.0	**93.5**	*98.4*	**94.0**	**92.2**
11 field	2.0%/2.0	*99.9*	*100.0*	*99.9*	*98.8*
	2.0%/1.0	*99.4*	*100.0*	*99.8*	*97.9*
	1.0%/1.0	***81.8***	*99.1*	*98.2*	**93.7**
Lung	2.0%/2.0	*96.4*	*96.6*	*98.1*	*96.1*
	2.0%/1.0	***89.0***	**93.5**	*96.7*	*95.7*
	1.0%/1.0	***55.2***	***72.5***	**90.7**	***88.2***
Head and neck	2.0%/2.0	*99.9*	*99.9*	*99.6*	*99.4*
	2.0%/1.0	*99.2*	*99.6*	*99.1*	*98.6*
	1.0%/1.0	*95.6*	**93.3**	*95.6*	**94.5**
Abdo	2.0%/2.0	*98.7*	*99.5*	*99.9*	*98.6*
	2.0%/1.0	*96.9*	*98.8*	*99.5*	*98.4*
	1.0%/1.0	***59.4***	***84.9***	**92.8**	*95.3*
Multi target	2.0%/2.0	*98.5*	*100.0*	*99.1*	*99.1*
	2.0%/1.0	*97.5*	*99.9*	*98.9*	*98.6*
	1.0%/1.0	***78.5***	*95.1*	*96.0*	*95.8*

### Clinical cases

**Fig. 10:**
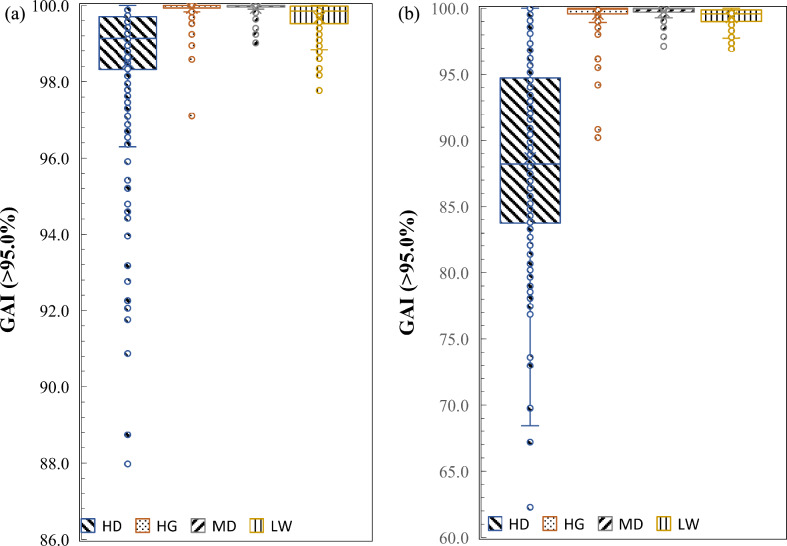
TQA GAI for 226 treatment plans using the following criteria **a** 3.0%/2.0 mm **b** 2.0%/2.0 mm

Retrospective gamma analysis (n = 226 clinical fractions) at 3.0%/2.0 mm reported 211 plan fractions (93.0%) achieving a gamma pass rate > 95.0% in high-dose regions (HD), and 100.0% of the plans achieving this pass rate in the other regions (HG, MD, LD) (Fig. 10a).

With a 2.0%/2.0 mm gamma criterion only 25.0% of the plans achieve gamma pass rate above 95.0% in the HD region. High pass rates were still observed in HG (98.0%) and remained at 100.0% in the MD and LD regions (Fig. 10b). Previous film-based PSQA reported that all fractions passed the 2.0%/2.0 mm criteria with average gamma (RGB triple-channel dosimetry) indices greater than 95.0%.

Further gamma analysis was performed on a single fraction of two particular sites, namely a pelvic node and a prostate + SV, as the pelvic node exhibited the lowest gamma pass rate in the HD region (62.3% at 2.0%/2.0 mm; 88.0% at 3.0%/2.0 mm), as did the prostate + SV (88.1% at 2.0%/2.0 mm; 99.2% at 3.0%/2.0 mm).

**Fig. 11:**
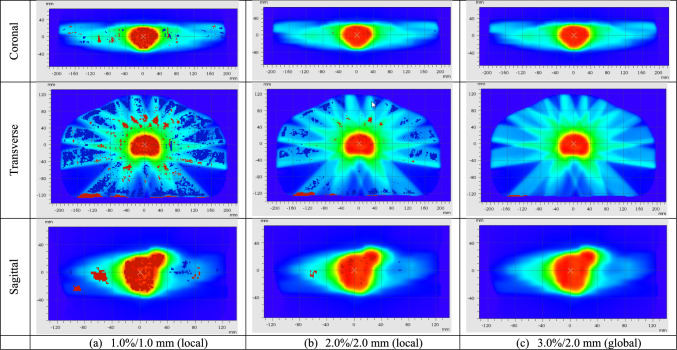
TQA–Monaco gamma comparison (1.0%/1.0 mm, 2.0%/2.0 mm and 3.0%/2.0 mm) heat map, for a prostate + SV, in the coronal, transverse and sagittal plane

**Fig. 12:**
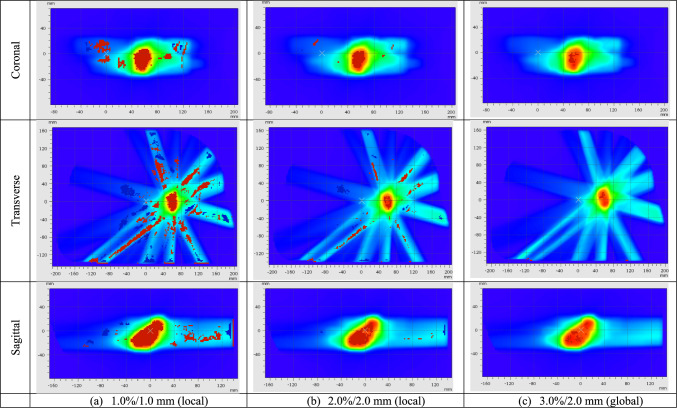
TQA–Monaco gamma comparison (1.0%/1.0 mm, 2.0%/2.0 mm and 3.0%/2.0 mm) heat map, for a pelvic lymph node, in the coronal, transverse and sagittal plane

Figures 11 and 12. show the gamma analysis dose maps and failing points for these. Failing points are indicated by dark red pixels (TQA hotter) and dark blue pixels (TQA colder). At 1.0%/1.0 mm (local), TQA calculated plans for these fractions consistently appear hotter within the PTV, while colder regions are observed in the surrounding low dose areas.

**Fig. 13:**
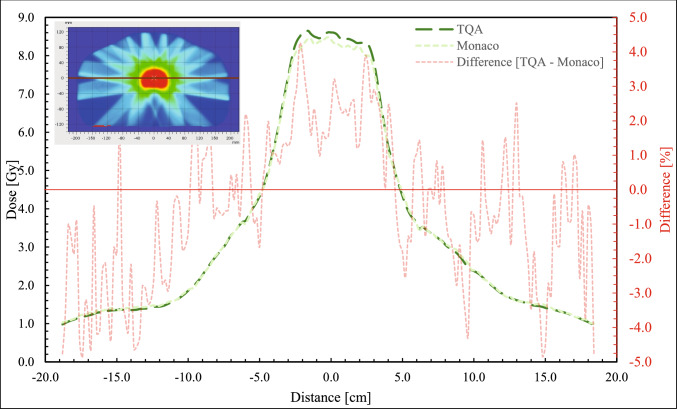
TQA and Monaco horizontal extracted dose profile through the PTV in the top left image. This image is taken from the prostate + SV case presented above in the transverse plane

To further illustrate dose discrepancies, a horizontal dose profile extracted from the transverse plane for the prostate fraction is shown in Fig. 13. Mean discrepancy of approximately 2.0% is observed within the PTV.

## Discussion

The TQA CCC algorithm has shown improved agreement with Monaco compared to the previously used Clarkson based MU2net [18]. The mean absolute dose difference of 0.4% between TQA and Monaco is within the MPPG5b recommended tolerance, indicating good agreement of TQA with Monaco under standard conditions [21]. The cross-plane and in-plane dose profiles presented in this work demonstrate that TQA v2.0.1.11 modelling of the static magnetic field is consistent with Monaco v.5.4.

The cryostat, the treatment couch and posterior coil contribute to the overall beam attenuation and all of these components are included in TQA dose calculations, Fig. 4 shows that for gantry angles of 0.0°, 30.0°–105.0° and 255.0°–345.0°, measured and Monaco normalised output values for a 5.0 cm × 5.0 cm field are in good agreement. Between Monaco-TQA the agreement of beams traversing the couch are within + 1.8% and − 1.2% and greatest discrepancies are observed at the couch edges (densest part of the couch) (MU2net reported discrepancies between 3.0 and 6.0%). All results are within 2.0%, TQA models the cryostat as well as the couch and posterior coil.

TQA and Monaco OFs at 133.5 cm SSD are within 1.0% and at 138.5 cm SSD are within 2.0% for field sizes ranging from 1.0 cm × 1.0 cm to 20.0 cm × 20.0 cm, which is within the recommended tolerance [21].

Off-axis point dose differences, are greatest for the 1.0 cm × 1.0 cm field size. Contributing factors would include correct modelling of lateral charged particle disequilibrium in the magnetic field. These differences are reduced as the field size is increased.

For the heterogeneous slab geometry, significant deviations occur near material interfaces due to the absence of explicit ERE modelling by TQA and this effect is more prominent as the field size is increased. The observed variation in dose in the vicinity of the interfaces investigated is similar to that previously reported for water–air-water slab geometry [25]. Away from interface regions the agreement between TQA and Monaco is acceptable with differences less than 2.0%. The absence of ERE modelling with TQA is a limitation that will impact on its use as an SDC in the presence of lung and air cavities [19].

Reducing the SU from 3.0 to 1.0% increases the Monaco calculation time by a factor of 4 and the effect on gamma comparisons is 7.3% in the HD region, and 0.2% in all other regions for the sampled plans in this work. The balance between grid size and SU needs to be considered to minimise calculation time without degrading the plan quality.

Of the nine Elekta sample plans, TQA and Monaco dose calculation comparison, showed that all apart from the lung plan achieve > 95% pass rate at 2.0%/2.0 mm.

Retrospective gamma analysis of clinical cases indicated acceptable passing rates using a gamma criterion of 3.0%/2.0 mm (global, 10.0% threshold). Further interrogation of two single fractions revealed systematic differences between TQA and Monaco at stricter criteria: 1.0%/1.0 mm (local) and 2.0%/2.0 mm (local). Specifically, TQA calculated higher doses within high dose regions relative to Monaco, which can be attributed to the energy fluence engine as well as MLC, leaf tip modelling and inter and intra leaf leakage which can be further optimised.

Further scrutiny of two treated fractions in the transverse plane (Figs. 11 and 2) revealed localised hot spots at the beam entry point (gantry angle 225.0° and 230.0° respectively). In this instance, an air gap was present between the patient’s external contour and the treatment couch, creating a low-density interface, generating ERE, which is currently not modelled in TQA. This discrepancy is localised and of minimal clinical relevance and does not significantly impact the overall gamma analysis.

Overall, comparing TQA and Monaco highlight the importance of comprehensive beam model validation, as the dose discrepancies observed could lead to potential clinical implications if interpreted incorrectly. Although previous film-based PSQA indicated robust agreement between Monaco and actual delivered doses, the systematic discrepancies noted here underline the sensitivity of the TQA algorithm to small calculation differences, especially under conditions of steep dose gradients and highly modulated treatment plans.

Considering the retrospective gamma analysis outcomes, the initial clinical gamma criterion (3.0%/2.0 mm) appears justified and appropriate, given the high passing rates (> 93.0%) in high-dose regions and universal passing in lower-dose regions. However, the significant reduction in passing rates in the HD region at 2.0%/2.0 mm gamma criteria highlights discrepancies between the algorithms. This suggests caution in adopting overly strict gamma thresholds without careful evaluation of the clinical context and recognition of inherent algorithmic limitations.

### Clinical workflow efficiency and practical considerations

At TUH, TQA complements, but does not replace, PSQA. A fraction-zero measurement establishes the baseline against the TPS. During ATP/ATS, TQA provides an independent online verification of the recalculated TPS dose; after delivery, each fraction receives a post-treatment PSQA measurement comparing the measured 2D dose with Monaco.

The integration of TQA into the clinical workflow has improved efficiency and enhanced plan verification capabilities. Notably, it has replaced the department’s previous secondary MU check software (MU2net), providing a more advanced, reliable and volumetric approach to secondary dose verification. In our experience MU2net produced DRP discrepancies > 5% resulting in iterative DRP reselection, without necessarily indicating a degraded plan. For the same plans TQA achieved > 95% pass rates at 3%/2 mm. This has improved QA reliability compared with historical point dose comparisons.

TQA requires minimal staff training due to its intuitive and user-friendly interface. Gamma analysis results are typically available within approximately one minute after export of adapted plans from Monaco, during this time the Radiation Therapists starts the motion monitoring scan to assess target motion within the PTV improving the efficiency of the treatment workflow. The system also allows users to perform on-the-fly recalculations with different gamma criteria for comparison with baseline values captured before the treatment course has commenced.

One of TQA’s key advantages is its ability to perform 3D volume-based gamma analysis, with structured reporting across four clinically relevant dose regions: HD, HG, MD and LD. Additionally, TQA supports gamma reporting to all delineated structures, facilitating more targeted evaluation of critical volumes such as PTVs and OARs.

The software also allows users to generate comprehensive QA reports and verify beam parameters of each adapted Monaco plan during the course of treatment, further supporting clinical quality assurance processes.

While the current version does not include gamma map visualization within the software, these features are expected in future versions, which will further enhance TQA’s analytical capabilities and utility in clinical practice.

It is important to note that initial beam model validation for TQA was performed outside the software environment. This process was data-intensive and required significant effort to compile and format the necessary datasets. Incorporating an integrated beam model validation tool within TQA would streamline implementation and support broader clinical adoption.

## Conclusion

TQA has demonstrated reliable performance as a secondary dose verification system for the Elekta Unity MR-Linac, supporting adaptive radiotherapy workflows through fast, volumetric gamma analysis. Its ease of use and minimal training requirements facilitate clinical integration, while its structured reporting and flexible evaluation criteria enhance plan assessment.

## Data Availability

All data relevant to this article can be made available upon request.
